# Interscalene Block Versus Pericapsular Nerve Block and Superficial Cervical Plexus Block for Arthroscopic Shoulder Surgery

**DOI:** 10.5812/aapm-165770

**Published:** 2025-11-29

**Authors:** Amr Arafa Elbadry, Marwa A. Abogabal, Laila Elahwal

**Affiliations:** 1 Anesthesiology, Surgical Intensive Care and Pain Medicine Department, Faculty of Medicine, Tanta University, Tanta, Egypt

**Keywords:** Interscalene Block, Pericapsular Nerve Block, Superficial Cervical Plexus Block, Shoulder Arthroscopy, Regional Anesthesia

## Abstract

**Background:**

Interscalene brachial plexus block (ISB) remains the gold standard for analgesia in arthroscopic shoulder surgery (ASS). However, ISB is associated with a higher incidence of hemidiaphragmatic paralysis (DP).

**Objectives:**

This study compares ultrasound-guided interscalene brachial plexus block (USG-ISB) with a combination of ultrasound-guided pericapsular nerve block (USG-PENB) and superficial cervical plexus block (SCPB) to evaluate analgesic efficacy and the incidence of DP.

**Methods:**

In this prospective, triple-blinded randomized trial, 42 American Society of Anesthesiologists (ASA) I - II patients undergoing elective ASS were randomized into two groups after induction of general anesthesia (GA): Group A (ISB, 10 mL 0.25% bupivacaine) or group B [pericapsular nerve block (PENB) 10 mL + SCPB 5 mL 0.25% bupivacaine]. Blocks were performed under ultrasound guidance. The primary outcome was the incidence of DP; secondary outcomes included pain scores, opioid consumption, pulmonary function, and patient satisfaction.

**Results:**

Compared with group A, group B demonstrated a delayed time to first request for rescue analgesia (13.24 vs. 8.38 hours; P < 0.001) and reduced 24-hour fentanyl consumption (135.71 vs. 192.86 mcg; P = 0.012). Pulmonary function was significantly better preserved in group B (P < 0.05). The incidence of DP was lower in group B (4.76% vs. 38.1%; P = 0.02). Pain scores at 6, 12, and 18 hours were also lower in group B (P < 0.05). Both groups showed no differences in hypotension, bradycardia, or patient satisfaction.

**Conclusions:**

The combination of PENB and SCPB provides analgesia non-inferior to ISB, while significantly reducing the incidence of DP and opioid requirements. For individuals at risk of respiratory impairment, this approach presents a lower-risk alternative without compromising pain control efficacy.

## 1. Background

With over half a million annual procedures, the high frequency of arthroscopic shoulder surgery (ASS) underscores its widespread adoption in orthopedic medicine (1). Advances in surgical techniques have broadened the spectrum of shoulder pathologies amenable to arthroscopic management (2, 3). Despite these developments, achieving effective analgesia during the immediate 24-hour post-surgical period remains a significant clinical challenge (4). This issue has become even more critical amid increasing concerns regarding opioid dependence among orthopedic patients (1). Inadequately managed postoperative pain contributes to delayed discharge, unexpected hospital readmissions, and compromised patient outcomes (5, 6). Therefore, facilitating improved patient recovery requires proficient pain control, which in turn enables shorter hospital stays and reduces healthcare costs associated with outpatient shoulder arthroscopy (7).

The nerve supply to the shoulder arises from both the cervical and brachial plexuses, with joint innervation predominantly provided by the anterior branches of the C5 and C6 cervical nerves (with a minor contribution from C7), while C3 and C4 primarily mediate cutaneous sensation via the superficial cervical plexus (8). As a result, regional anesthetic techniques targeting these neural structures can substantially enhance postoperative pain management (9). Interscalene brachial plexus block (ISB) offers effective pain relief but is commonly associated with hemidiaphragmatic paralysis (DP), thereby limiting its applicability in patients with pulmonary compromise. This has led to the investigation of alternative approaches, such as suprascapular and pericapsular nerve blocks (PENB), with or without superficial cervical plexus block (SCPB), to better preserve pulmonary function. This limitation has prompted the exploration of alternative techniques (10), including suprascapular block (11) and PENB (12, 13). The latter specifically targets articular branches innervating the glenohumeral joint (14), without inducing motor blockade or pulmonary complications.

To the best of our knowledge, no prior research has directly evaluated the use of combined PENB and SCPB as an alternative to ISB in patients undergoing ASS. This gap in the literature provided a strong rationale for conducting the present study.

## 2. Objectives

Our study aims to compare ultrasound-guided interscalene brachial plexus block (USG-ISB) and ultrasound-guided pericapsular nerve block (USG-PENB) combined with SCPB in patients undergoing ASS. We hypothesize that this combination may offer comparable analgesia while reducing the risk of DP.

## 3. Methods

A prospective, triple-blinded, randomized, controlled study, registered under NCT05768009 and ethically approved (ID: 36264PR38/1/23), evaluated 42 adult patients classified as American Society of Anesthesiologists (ASA) (15) physical status I or II, aged 18 - 65 years, and undergoing elective ASS at Tanta University hospitals, Egypt, from January to June 2023. All participants provided informed written consent. Patients outside the specified age range were excluded, as were those who refused to participate or had peripheral neuropathy, ASA classification greater than II, bleeding diathesis, chronic chest diseases affecting pulmonary function, cutaneous infection at the injection site, pacemakers, recent history of anticoagulants, pregnancy, atrioventricular block, or known allergy to local anesthetics.

### 3.1. Randomization and Blindness

Participants were equally allocated to two groups using sealed opaque envelopes containing computer-generated random numbers (randomizer). After induction of general anesthesia (GA), group A received USG-ISB using 10 mL of 0.25% bupivacaine, while group B received a combination of USG-PENB (10 mL of 0.25% bupivacaine) and SCPB (5 mL of 0.25% bupivacaine). The study was conducted in a triple-blinded manner. Patients, surgeons, and postoperative assessors were blinded to group allocation. The anesthesiologist performing the block did not participate in intraoperative management or postoperative data collection, thereby maintaining the integrity of blinding.

### 3.2. Preoperative Assessment and Preparation

Comprehensive preoperative assessments were conducted for all participants, including detailed patient histories, thorough clinical evaluations, radiological shoulder imaging, and routine laboratory analyses. Participants were educated on the use of an eleven-point Numerical Rating Scale (NRS) (16) for pain assessment, ranging from 0 (no pain) to 10 (worst pain imaginable). Ultrasound imaging was performed using a Mindray system (Shenzhen, China) with a frequency range of 10 - 15 MHz and a 2 - 4 cm depth setting. A curvilinear probe was used for scanning via a low intercostal or subcostal approach, while a linear probe was employed for various block procedures.

### 3.3. Assessment of Preoperative Diaphragmatic Function and Pulmonary Parameters

Preoperative assessment of diaphragmatic motion was conducted using ultrasound in both B-mode and M-mode configurations. Patients were positioned supine and underwent scanning with a curvilinear transducer applied via a low intercostal or subcostal technique, utilizing the liver on the right or the spleen on the left as acoustic windows. Diaphragmatic displacement during standard breathing cycles was evaluated to exclude pre-existing motility impairments and was followed by the administration of 'sniff' and 'sigh' maneuvers. In the sniff maneuver, diaphragmatic motion was observed from an expiratory baseline during rapid nasal inhalations. In contrast, the sigh maneuver measured the diaphragmatic motion spectrum from resting expiratory to maximal inspiratory effort. Inspiratory caudad motion of the diaphragm was denoted as positive, whereas cephalad, paradoxical displacement was labeled negative. Each parameter was measured twice, with the results subsequently averaged. The extent of diaphragmatic movement was recorded in centimeters.

Respiratory function assessments were performed with participants in a seated position using a portable spirometer. The following parameters were measured: Forced vital capacity (FVC), forced expiratory volume in one second (FEV1), the FEV1/FVC ratio, and airflow rate expressed in liters per second. Three separate readings were obtained from each patient, and the mean value was calculated.

Upon arrival in the operating room, a peripheral intravenous (IV) catheter was inserted into a suitable forearm vein on the limb opposite to the operative field. Standard monitoring was initiated for all subjects, including pulse oximetry, a temperature sensor, noninvasive arterial pressure measurement, electrocardiographic leads, and capnography. Prior to the intervention, each participant received 2 mg of midazolam and 50 mcg of fentanyl.

### 3.4. The Technique of General Anesthesia

The GA was induced by IV administration of propofol at a dose of 2 mg/kg, fentanyl at 2 mcg/kg, and cisatracurium at 0.15 mg/kg. Endotracheal intubation was then performed using a tube of appropriate diameter, followed by the initiation of controlled mechanical ventilation, set to deliver a tidal volume of 6 - 8 mL/kg and a positive end-expiratory pressure of 5 cm H_2_O. Anesthesia was maintained with a balanced mixture of oxygen and air (50%-50%) and isoflurane at a concentration of 1 - 1.5%. Ongoing muscle relaxation was achieved with intermittent doses of cisatracurium at 0.03 mg/kg. Ventilatory parameters were adjusted to maintain end-tidal carbon dioxide levels at approximately 35 mmHg. If heart rate (HR) and/or mean arterial pressure (MAP) exceeded baseline values by more than 20%, additional IV fentanyl was administered.

### 3.5. Block Procedures

All nerve blocks were performed under ultrasound guidance (USG) and strict aseptic conditions following appropriate skin preparation and draping. Performing the blocks after induction of GA was chosen to maximize patient comfort and immobility during USG procedures, a technique previously reported as safe in other trials, with no complications observed in our cohort.

#### 3.5.1. Ultrasound-Guided Interscalene Brachial Plexus Block

The ISB was performed with the patient in the supine position, head slightly elevated and rotated away from the side to be blocked. The ultrasound probe was initially placed near the clavicular midline at the level of the cricoid cartilage, then moved laterally to visualize the carotid artery and internal jugular vein beneath the sternocleidomastoid muscle. Further lateral movement revealed the anterior scalene muscle beneath the lateral margin of the sternocleidomastoid. At this point, a groove typically containing hypoechoic neural elements was identified. Ten milliliters of 0.25% bupivacaine were injected into the scalene groove, encasing the nerve roots.

#### 3.5.2. Ultrasound-Guided Pericapsular Nerve Block

The patient’s arm was abducted to 45 degrees and externally rotated. The ultrasound transducer was positioned longitudinally between the coracoid process and the humeral head. Once the humeral head, subscapularis tendon, and superficial deltoid muscle were visualized, a 50-mm sonovisible needle was introduced using an in-plane technique. The needle tip was directed between the deltoid muscle and subscapularis tendon, where 10 mL of 0.25% bupivacaine was injected.

#### 3.5.3. Ultrasound-Guided Superficial Cervical Plexus Block

Patients were placed supine, with the ipsilateral shoulder relaxed and slightly elevated, and the head turned to the opposite side. A marker was positioned at the midpoint of the posterior border of the clavicular head of the sternocleidomastoid muscle, approximately at the level of the cricoid cartilage and 3 - 4 cm above the clavicle. The ultrasound probe was oriented transversely at this marked site. After identifying the sternocleidomastoid muscle, the probe was moved posteriorly until the posterior edge of the muscle was centered on the image. The investing fascia and prevertebral fascia were identified from superficial to deeper planes. Using a long-axis in-plane approach, a sonovisible needle was inserted from the lateral border of the sternocleidomastoid muscle. Under USG, the needle tip was confirmed to be between the deep layer of the investing fascia and the superficial layer of the prevertebral fascia near the sternocleidomastoid border. After negative aspiration for blood or cerebrospinal fluid, 5 mL of 0.25% bupivacaine was injected.

After surgery, inhalational anesthesia was discontinued. Once adequate spontaneous respiration was observed, neuromuscular blockade was reversed with a combination of neostigmine (0.05 mg/kg) and atropine (0.01 mg/kg). Extubation was performed when standard extubation criteria were fulfilled.

### 3.6. Post-operative Assessment

After completion of the ASS, all patients were transferred to the Post-anesthesia Care Unit (PACU) and subsequently to the inpatient ward for continued monitoring. The HR and MAP were recorded in the PACU at 2, 4, 6, 12, 18, and 24 hours postoperatively. Post-operative pulmonary function, assessed by serial diaphragmatic ultrasound and spirometry, was evaluated in the PACU and 24 hours after surgery. The DP was defined as a diaphragmatic motion decrease of greater than 75%, immobility, or paradoxical movement. A motion reduction between 25% and 75% indicated partial DP, while a reduction of less than 25% was classified as no paralysis (4).

Post-operative pain was assessed using the NRS, with scores recorded in the PACU at 2, 4, 6, 12, 18, and 24 hours after surgery. Post-operative analgesia consisted of IV acetaminophen (1 g every 8 hours) and IV ketorolac (30 mg every 12 hours). For breakthrough pain (NRS > 3), IV fentanyl (25 - 50 mcg) was administered as rescue analgesia. The time until the first episode of breakthrough pain and total fentanyl consumption were documented. Patient satisfaction with pain management was evaluated using a five-point Likert scale (17), ranging from 0 to 4 (weak, medium, good, very good, and excellent, respectively).

The primary outcome measure was the incidence of post-operative DP. Secondary outcomes included changes in intraoperative hemodynamics, post-operative pain scores, time until the first request for rescue analgesia, percentage of cases requiring rescue analgesia, total post-operative opioid consumption, incidence of other block-related complications, and satisfaction with the anesthetic technique.

### 3.7. Sample Size Calculation

Based on an extensive literature review, no previous studies had directly compared these specific block regimens. A pilot study comparing the two regimens (10 patients per group) found the incidence of DP to be 30% in the ISB group versus 0% in the combined PENB and SCPB group. Accordingly, 21 cases were required in each study group to achieve 80% power at a significance level of 0.05.

### 3.8. Statistical Analysis

Statistical analysis was performed using SPSS version 27 (IBM^©^, Armonk, NY, USA). Data normality was assessed using the Shapiro-Wilks test and histogram analysis. For parametric quantitative data, means and standard deviations (SDs) were calculated and compared using the unpaired student *t*-test. Non-parametric quantitative variables were reported as medians and interquartile ranges (IQRs), with statistical significance determined using the Mann-Whitney test. Qualitative variables were expressed as frequencies and percentages, and analyzed with either the chi-square or Fisher’s exact test, as appropriate. A two-tailed P-value of ≤ 0.05 was considered statistically significant.

## 4. Results

**Figure 1.**
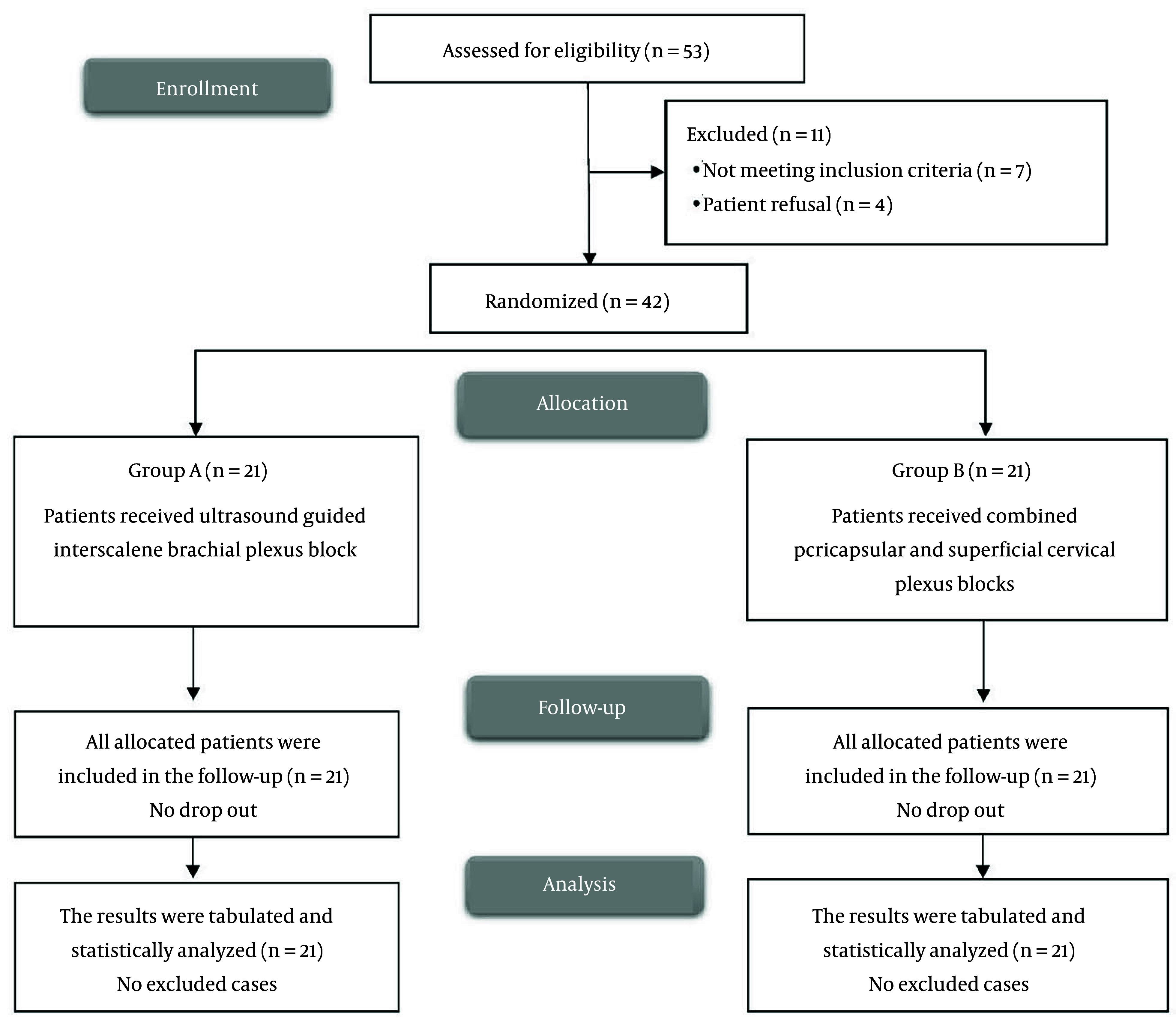
CONSORT flowchart of the enrolled patients

Eligibility screening in this research identified 53 patients, of whom 7 did not meet the inclusion criteria and 4 declined participation. The remaining patients were randomly assigned to two equal groups of 21 participants each. All enrolled cases were followed up and included in the statistical analyses (Figure 1). Demographic data and duration of surgery were comparable between the two groups (Table 1). All arthroscopic procedures were of similar complexity (primarily rotator cuff repair and subacromial decompression), with no significant differences in surgical time between the groups.

**Table 1.** Demographic Data and Duration of Surgery of the Studied Groups ^a^

Variables	Group A (N = 21)	Group B (N = 21)	P-Value	RR/Mean Difference (95% CI)
**Age (y)**	49.24 ± 10.02	47.86 ± 13.93	0.714	1.38 (-6.19 to 8.95)
**Sex**			0.747	1.08 (0.69 to 1.69)
Male	14 (66.67)	13 (61.9)		
Female	7 (33.33)	8 (38.1)		
**Weight (kg)**	84.29 ± 15.96	81.52 ± 12.58	0.537	2.76 (-6.2 to 11.72)
**Height (cm)**	167.33 ± 7.71	166.1 ± 6.76	0.583	1.24 (-3.28 to 5.76)
**Body Mass Index (kg/m** ^**2**^ **)**	30.12 ± 5.54	29.63 ± 4.87	0.764	0.49 (-2.77 to 3.74)
**ASA physical status**			0.739	1.07 (0.71 to 1.61)
I	15 (71.43)	14 (66.67)		
II	6 (28.57)	7 (33.33)		
**Duration of surgery (min)**	44.76 ± 11.99	41.43 ± 7.61	0.288	3.33 (-2.93 to 9.59)

Abbreviations: RR, relative risk; CI, confidence interval; ASA, American Society of Anesthesiologists.

^a^ Values are expressed as mean ± standard deviation (SD) or No. (%).

**Figure 2.**
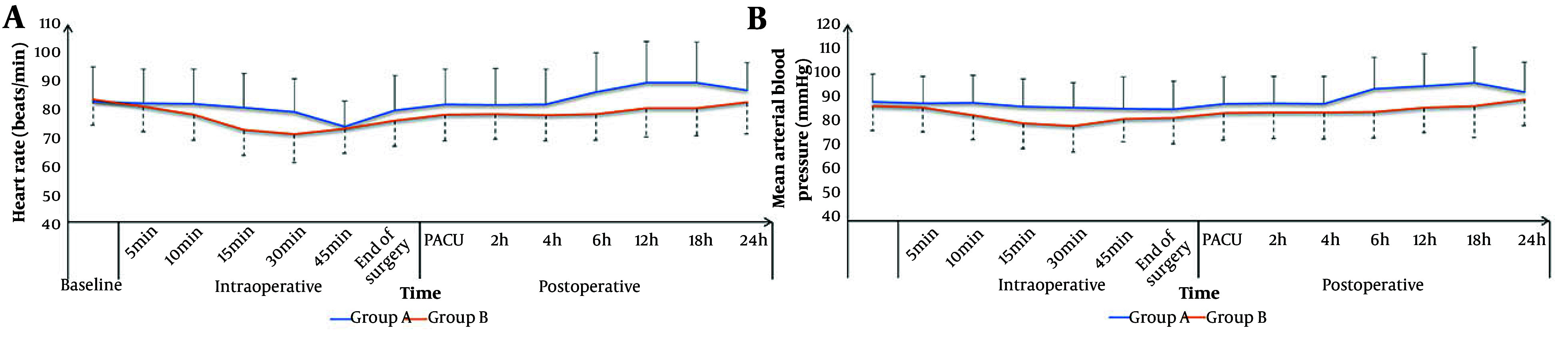
A, heart rate (HR); and B, mean arterial pressure (MAP) changes of the studied groups

The HR and MAP were not significantly different between the groups at baseline; 5, 10, and 15 minutes intraoperatively; in the PACU; and at 2, 4, and 24 hours postoperatively. The HR and MAP were significantly lower in group B compared to group A at 30 and 45 minutes intraoperatively and at 6, 12, and 18 hours postoperatively (P < 0.05, Figure 2). 

A significant prolongation in the time to the first request for rescue analgesia and a reduction in 24-hour fentanyl consumption were observed in group B compared to group A (P < 0.001 and P = 0.012, respectively), accompanied by higher FVC and FEV1 values (P < 0.05), although the FEV1/FVC ratio did not differ between the groups (Table 2). 

**Table 2.** Time to the First Request of Rescue Analgesia, Fentanyl Consumption, and Pulmonary Function of the Studied Groups ^a^

Variables	Group A (N = 21)	Group B (N = 21)	P-Value	Mean Difference (95% CI)
**Time for the first request of rescue analgesia (h)**	8.38 ± 1.47	13.24 ± 2.49	< 0.001 ^b^	-4.86 (-6.13 to -3.58)
**Total dose of fentanyl consumption in the first 24 hours (mcg)**	192.86 ± 77.11	135.71 ± 63.53	0.012 ^b^	57.14 (13.08 to 101.21)
**Pulmonary function tests**				
FVC (%)	86.38 ± 10.91	92.62 ± 7.41	0.036 ^b^	-6.24 (-12.05 to -0.42)
FEV1 (%)	85.57 ± 14.6	93.76 ± 8.54	0.032 ^b^	-8.19 (-15.65 to -0.73)
FEV1/FVC	0.99 ± 0.1	1.02 ± 0.09	0.383	-0.03 (-0.09 to 0.03)

Abbreviations: FVC, forced vital capacity; FEV1, forced expiratory volume.

^a^ Values are presented as mean ± standard deviation (SD).

^b^ Significant P-value < 0.05.

The NRS pain score showed no significant differences between the groups in the PACU and at 2, 4, and 24 hours; however, it was significantly lower in group B than in group A at 6, 12, and 18 hours postoperatively (P < 0.05, Table 3). 

**Table 3.** Numerical Rating Scale Score of the Studied Groups ^a^

Variables	Group A (N = 21)	Group B (N = 21)	P-Value	Median Difference (95% CI)
**PACU**	0 (0 - 1)	1 (0 - 1)	0.218	0 (0 to 1)
**2 h**	1 (0 - 1)	1 (0 - 1)	0.542	0 (-1 to 0)
**4 h**	1 (1 - 2)	1 (1 - 2)	0.156	0 (-1 to 0)
**6 h**	2 (2 - 3)	2 (1 - 2)	0.008 ^b^	-1 (-1 to 0)
**12 h**	3 (2 - 4)	2 (1 - 2)	0.010 ^b^	-1 (-1 to 0)
**18 h**	3 (2 - 4)	2 (1 - 3)	0.028 ^b^	-1 (-2 to 0)
**24 h**	3 (2 - 4)	3 (2 - 4)	0.571	0 (-1 to 1)

Abbreviations: CI, confidence interval; PACU, Post-anesthesia Care Unit.

^a^ Values are presented as median [interquartile range (IQR)].

^b^ Significant P-value < 0.05.

The incidence of post-operative DP was 38.1% (8 patients) in group A and 4.76% (1 patient) in group B. The incidence of post-operative DP was significantly higher in group A compared to group B (P = 0.020), with a relative risk (RR) of 8 (95% CI: 1.09 - 58.46). Patient satisfaction, as well as the incidences of bradycardia, hypotension, and postoperative nausea and vomiting (PONV), showed no significant differences between the groups (Table 4). 

**Table 4.** Complications and Patient Satisfaction of the Studied Groups ^a^

Variables	Group A (N = 21)	Group B (N = 21)	P-Value	RR/(95%CI)
**Complications**				
The incidence of post-operative diaphragmatic paralysis	8 (38.1)	1 (4.76)	0.020	8 (1.09 to 58.46)
Bradycardia	2 (9.52)	3 (14.29)	1	0.67 (0.12 to 3.59)
Hypotension	3 (14.29)	5 (23.81)	0.696	0.6 (0.16 to 2.2)
Post-operative nausea and vomiting	4 (19.05)	2 (9.52)	0.662	2 (0.41 to 9.77)
Respiratory depression	0 (0)	0 (0)	-	-
**Patient satisfaction**			0.848	-
Excellent	6 (28.57)	8 (38.1)		
Very good	4 (19.05)	4 (19.05)		
Good	5 (23.81)	6 (28.57)		
Medium	4 (19.05)	2 (9.52)		
Weak	2 (9.52%)	1 (4.76%)		

Abbreviations: RR, relative risk; CI, confidence interval.

^a^ Values are expressed as No. (%).

## 5. Discussion

This study compared the combination of PENB and SCPB with ISB for ASS. The main findings were that PENB combined with SCPB preserved pulmonary function and reduced the incidence of DP, while providing analgesia comparable to that of ISB. Differences in pain scores were modest and primarily observed between 6–18 hours postoperatively.

Our results showed similar hemodynamic parameters between both techniques at baseline and during the early intraoperative period. However, group B demonstrated significantly better hemodynamic stability during the intermediate intraoperative and postoperative periods compared to group A. These findings are consistent with those of Diab et al. (18), who reported minimal hemodynamic alterations when using combined supraclavicular block (SCB) and SCPB for shoulder surgeries, with no significant changes in MAP or HR throughout the perioperative period. This enhanced stability may be explained by the findings of Ibrahim Mohammed Khater et al. (19), who documented superior hemodynamic stability with regional anesthesia techniques compared to GA alone.

The most clinically significant advantage of the combined block technique was its superior analgesic profile. Consistent with our findings, Kilbasanli and Kacmazb (20) reported reduced analgesic requirements and lower pain scores when ISB was supplemented with SCPB, compared to GA alone. Similarly, Dabi et al. (21) observed a notable extension in analgesic duration when combining SCB and SCPB for shoulder surgeries. The enhanced efficacy of combined techniques likely results from a more comprehensive blockade of the shoulder joint’s innervation. As Kupeli and Kara (12) noted, although ISB effectively targets the brachial plexus, techniques such as PENB provide supplementary coverage of the sensory innervation to the glenohumeral joint, without causing motor blockade or respiratory impairment.

While both techniques provided comparable pain control in the immediate postoperative period, the combined approach showed similar analgesia with modest advantages during the intermediate postoperative phase, before converging with ISB efficacy at 24 hours. This pattern suggests that the combined technique offers extended intermediate-term analgesia, precisely when the efficacy of traditional ISB typically begins to diminish. Galluccio et al. (13) likewise reported sustained analgesic efficacy with shoulder anterior capsular blocks, used either alone or in combination with other techniques, supporting the notion that targeting the joint capsule yields effective and durable pain relief.

A primary concern with ISB is its association with phrenic nerve paralysis and subsequent compromise of pulmonary function. Our results demonstrate a significant advantage of the combined PENB and SCPB techniques in preserving respiratory mechanisms.

These findings are consistent with the growing body of evidence seeking alternatives to traditional ISB that minimize respiratory complications. Jo et al. (22) showed that upper trunk block significantly reduced the incidence of complete DP compared to ISB (5.9% vs. 41.7%, P < 0.001), while maintaining comparable analgesic efficacy. Similarly, Kang et al. (23) reported that superior trunk block led to a dramatically lower incidence of complete DP compared to ISB (5.3% vs. 72.5%), while preserving spirometry values. Aliste et al. (24) documented a marked reduction in hemi-DP with modified SCB compared to ISB (9% vs. 95%, P < 0.001), with equivalent post-operative analgesia.

Our findings contribute to this evolving paradigm by demonstrating that combined PENB and SCPB represent another viable strategy for minimizing respiratory compromise while maintaining effective analgesia. The preservation of pulmonary function observed in our study is of particular clinical significance for patients with pre-existing respiratory conditions, for whom traditional ISB may be contraindicated. As Kupeli and Kara (12) emphasized, techniques such as PENB are specifically designed to address this limitation of ISB by selectively targeting the sensory branches of the glenohumeral joint without inducing respiratory impairment.

The most significant advantage of the combined PENB and SCPB technique was its substantially improved safety profile and reduced incidence of DP, especially for patients with compromised pulmonary function. Our findings align with the accumulating evidence documenting the high incidence of DP associated with ISB. Aliste et al. (24) reported a 95% incidence of hemi-DP with traditional ISB, while Kang et al. (23) observed complete DP in 72.5% of ISB patients. The substantially lower incidence observed with the combined PENB and SCPB (4.76%) in our study is comparable to the rates reported with other alternative techniques, such as the 5.9% incidence with upper trunk block reported by Jo et al. (22) and the 5.3% incidence with superior trunk block documented by Kang et al. (23). Han et al. (25) also reported a low incidence of hemi-DP (12%) with combined cervical plexus and costoclavicular blocks for ASS, with effective post-operative pain control and no neurological deficits.

Importantly, other complications — including bradycardia, hypotension, and PONV — showed no significant differences between groups, and no instances of respiratory depression were observed in either group. Similarly, Diab et al. (18) found no major complications with combined SCB and SCPB for shoulder surgeries. The favorable safety profile of combined regional techniques likely results from their more targeted approach to shoulder innervation (12).

Patient satisfaction was comparable between the two groups but tended to be somewhat higher in the combined block technique, further supporting the clinical value of this approach. Musso et al. (26) likewise reported high patient satisfaction with multimodal regional anesthesia techniques for ASS, with all patients expressing satisfaction with their anesthesia experience.

The small sample size and single-center design limit the generalizability of this study. Follow-up was restricted to 24 hours, precluding assessment of long-term outcomes. Although surgical procedures were comparable, minor variations in technique may have influenced the results. Additionally, the anesthesiologist performing the block was not blinded, which could introduce bias.

In conclusion, combining PENB and SCPB provided analgesia comparable to that of ISB, with a markedly lower incidence of diaphragmatic paralysis (4.76% vs. 33.33%) and reduced opioid use. The combination group also better preserved pulmonary function (FVC and FEV1). This approach offers effective pain control while mitigating ISB-associated respiratory risks, making it a promising alternative for ASS, particularly in patients with pulmonary vulnerability. Future large-scale studies are warranted to confirm these observations and to evaluate long-term outcomes.

## Data Availability

Data are available upon reasonable request from the corresponding author.

## Clinical Trial Registration Code:

NCT05768009.
